# Onatasertib re-sensitizes refractory nasopharyngeal carcinoma to immunotherapy: a Case Report

**DOI:** 10.3389/fphar.2026.1614224

**Published:** 2026-07-17

**Authors:** Peipei Zhang, Zhanpeng Zhang, Jun Liu, Yanfei Cui, Daming Qin, Lin Lai, Ying Xiang, Li Wang, Dezhong Li, Kai Luo, Chuying Huang

**Affiliations:** 1 Hubei Minzu University Affiliated Enshi Clinical Medical School, the Central Hospital of Enshi Tujia and Miao Autonomous Prefecture, Enshi, China; 2 Hubei Provincial Key Lab of Selenium Resources and Bioapplications, Enshi, China; 3 Hubei Selenium and Human Health Institute, The Central Hospital of Enshi Tujia and Miao Autonomous Prefecture, Enshi, China; 4 Cancer Center, The Central Hospital of Enshi Tujia and Miao Autonomous Prefecture, Enshi, China; 5 College of Biological and Food Engineering, Hubei Minzu University, Enshi, China

**Keywords:** mTOR inhibitor, nasopharyngeal carcinoma, onatasertib, PD-1 blockade, recurrent or metastatic cancer, resistance to immunotherapy

## Abstract

The standard first-line treatment for patients with recurrent or metastatic nasopharyngeal carcinoma (R/M-NPC) is currently a combination of a programmed cell death protein-1 (PD-1) blockade and gemcitabine-cisplatin. However, effective options beyond second-line settings are limited, and a recommended regimen is currently unavailable. This report presents a case study highlighting the significant efficacy of onatasertib, a dual mechanistic target of rapamycin (mTOR) 1/2 inhibitor, in overcoming resistance to immunotherapy in patients with R/M-NPC. A 28-year-old female patient who was diagnosed with nasopharyngeal carcinoma (NPC) experienced tumor relapse following progression on multiple lines of therapy, including immunotherapy. Subsequently, the patient participated in a phase I/II study (TORCH-2) investigating the combination of onatasertib and toripalimab in patients with advanced solid tumors. The patient achieved a nearly complete remission (CR) and experienced a progression-free survival (PFS) of 16 months. This unique case report highlights the efficacy of onatasertib, a dual mTOR1/2 inhibitor, in treating patients with R/M-NPC who have developed resistance to immunotherapy. The occurrence of a grade 2 serious adverse event (AE) underscores the importance of monitoring patients closely during treatment and the need to adjust dosages when necessary to minimize AEs. Meanwhile, it is important to note that the small sample size in this case is a significant limitation. Therefore, further randomized clinical trials are required to evaluate the efficacy and safety of this combination treatment in patients with acquired immunotherapy resistance and R/M-NPC.

## 1 Introduction

Nasopharyngeal carcinoma (NPC) is an aggressive malignant head and neck tumour that exhibits a remarkably skewed global distribution, with the highest incidence concentrated in East and Southeast Asia (Ch et al., 2019). This distinct geographical distribution is strongly associated with endemic risk factors, particularly Epstein-Barr virus (EBV) infection and specific genetic predispositions prevalent in these regions (Chang et al., 2021). China bears a particularly heavy burden, with over 51,000 new cases and an age-standardized incidence rate of 2.4 per 100,000 population (Su et al., 2024). Despite advances in modern treatment, a subset of patients still experience disease recurrence and ultimately die from distant metastasis, locoregional relapse, or both. (Jiang et al., 2024). For those who develop recurrent or metastatic (R/M) NPC, the gemcitabine plus cisplatin regimen has long been the standard first-line treatment, and the addition of programmed cell death protein-1 (PD-1) inhibitors to standard chemotherapy results in longer progression-free survival (PFS) and overall survival (OS) benefits (Mai et al., 2023). However, there is currently no standard second-line treatment for patients with pretreated R/M-NPC. Although immunotherapy has been approved as a subsequent-line treatment for R/M-NPC, primary and acquired resistance to anti-PD-1 therapy remains a major clinical challenge.

The phosphatidylinositol 3-kinase (PI3K), protein kinase B (AKT) and mechanistic target of rapamycin (mTOR) (PAM) pathway is a central regulator of tumor immune evasion and resistance to immunotherapy. On the one hand, hyperactivation of PAM signaling in cancer cells upregulates programmed death-ligand 1 (PD-L1) expression (Guo et al., 2025) and induces other immune checkpoint molecules such as B7-H3/CD276, which suppresses cytotoxic T-cell activity and fosters an immunosuppressive tumor microenvironment (Liu et al., 2023). On the other hand, mTOR signaling also profoundly shapes the host immune system: it controls T-cell activation, differentiation, and metabolism. Specifically, CD28 co-stimulation promotes glycolysis via the PAM axis, whereas PD-1 and CTLA-4 inhibit this metabolic reprogramming, ultimately leading to T-cell exhaustion (Wang S. et al., 2024). These mechanistic insights provide a strong rationale for targeting mTOR to overcome acquired resistance to PD-1 blockade in R/M-NPC. Furthermore, aberrant mTOR activation in tumor cells promotes the recruitment of immunosuppressive tumor-associated macrophages and induces aberrant sialylation of PD-L1, both of which further impair immune recognition (Liu et al., 2023; Lin et al., 2023; Guan et al., 2025). Collectively, these mechanistic insights establish mTOR as a critical nexus linking intrinsic tumor signaling to extrinsic immune evasion, thereby providing a robust rationale for targeting mTOR to overcome acquired resistance to PD-1 blockade in R/M-NPC.

Based on this rationale, we hypothesized that combining a dual mTOR complex (mTORC) 1/2 inhibitor with PD-1 blockade could re-sensitize immunotherapy-refractory NPC. Fortunately, we found that mTOR inhibitor therapy represents a new strategy for successfully overcoming immunotherapy resistance. Herein, we report a case of recurrent NPC with acquired resistance to immunotherapy, which was successfully treated with the dual mTORC1/2 inhibitor onatasertib, providing new insights into overcoming immunotherapy resistance.

## 2 Case description

**FIGURE 1:**
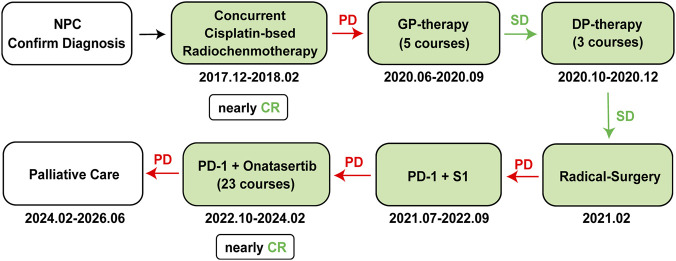
Timeline illustrating the whole treatment of toripalimab and onatasertib.

A 28-year-old female patient who was diagnosed with Epstein-Barr encoding region-positive nonkeratinizing undifferentiated NPC (T3N2M0, American Joint Commission on Cancer 7th Edition), achieved a nearly complete remission (CR) following concurrent cisplatin-based radiochemotherapy (Figure 1), as evidenced by the planning gross tumor volume of the nasopharynx (PGTVnx): 70 Gy/32 F, planning gross target volume of the metastatic lymph node (PGTVnd): 68 Gy/32 F, planning clinical target volume 1 (PCTV1): 60 Gy/32 F, planning clinical target volume 2 (PCTV2): 54 Gy/32 F. After 27 months, local recurrence was confirmed via magnetic resonance imaging (MRI) and nasopharyngeal endoscopic biopsy. The patient received 5 cycles of first-line gemcitabine plus cisplatin therapy (gemcitabine: 1,500 mg/m^2^ on day 1 and day 8, cisplatin: 35 mg/m^2^ from day 1 to day 3), resulting in stable disease. Subsequently, 3 cycles of second-line DP therapy were administered with docetaxel and lobaplatin (docetaxel: 110 mg/m^2^, lobaplatin: 40 mg/m^2^), but the clinical efficacy was stable. Radical surgery for NPC was then performed; however, MRI revealed local recurrence 5 months after surgery. As there is no standard third-line therapy regimen for R/M-NPC patients who have failed previous treatments, a combination of immunotherapy with toripalimab (200 mg, intravenous infusion) and tegafur/gimeracil/oteracil (40 mg twice daily from day 1 to day 14) was administered. Unfortunately, tumor progression was clinically recognized 14.6 months later despite this treatment approach. The patient was then enrolled in a phase I/II study investigating onatasertib, a dual TORC1/2 inhibitor combined with the PD-1 antibody toripalimab in advanced solid tumors (TORCH-2).

**FIGURE 2:**
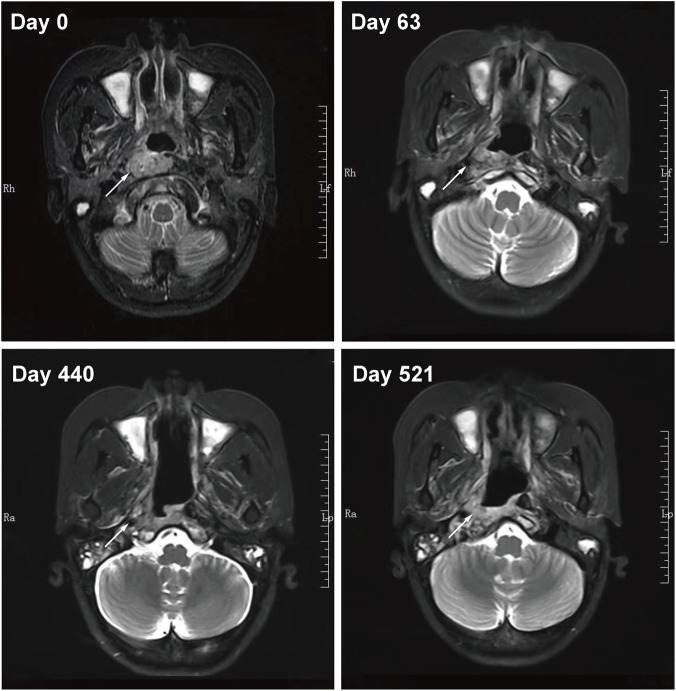
Magnetic resonance imaging illustrating changes in tumor size (white arrow) with onatasertib and toripalimab treatment.

The patient received 23 cycles of treatment with toripalimab and onatasertib. Toripalimab was administered via intravenous infusion at a fixed dose of 240 mg once every 3 weeks (Q3W), and onatasertib was given orally at 15 mg once daily (QD). Surprisingly, after two cycles of onatasertib, MRI showed a near-CR (Figure 2). On 12 June 2023, the patient reported a grade 2 AE characterized by unexpected weight loss (>10%). Consequently, the dosage of onatasertib was reduced from 15 mg/kg to 10 mg/kg. The AE persisted for 7 treatment cycles, and the patient’s body weight returned to baseline level (Table 1).

**TABLE 1:** Summary of the patient’s body weight changes and corresponding adverse event grades from baseline through the end of treatment.

Adverse event related to weight loss	
Baseline level	70 kg
Cycle 12 (2023.6.12)	62 kg (grade 2)
Cycle 13 (2023.7.5)	62 kg (grade 2)
Cycle 14 (2023.7.26)	62 kg (grade 2)
Cycle 15 (2023.8.16)	59 kg (grade 2)
Cycle 16 (2023.9.6)	61 kg (grade 2)
Cycle 17 (2023.9.27)	62 kg (grade 2)
Cycle 18 (2023.10.19)	63 kg (grade 2)
Cycle 19 (2023.11.9)	66 kg (grade 1)
Cycle 20 (2023.11.29)	66 kg (grade 1)
Cycle 21 (2023.12.20)	65 kg (grade 1)
Cycle 22 (2024.1.10)	64 kg (grade 1)
Cycle 23 (2024.1.31)	64 kg (grade 1)
End of study visit (2024.2.21)	59 kg (grade 2)

A recent study demonstrated that a negative status of EBV DNA is positively correlated with a greater objective response to camrelizumab and longer median PFS in patients with previously treated R/M NPC (Yang et al., 2021a). Notably, during the treatment period in our patient, the EBV DNA copy number decreased to an undetectable level and remained so throughout the combination therapy.

**FIGURE 3:**
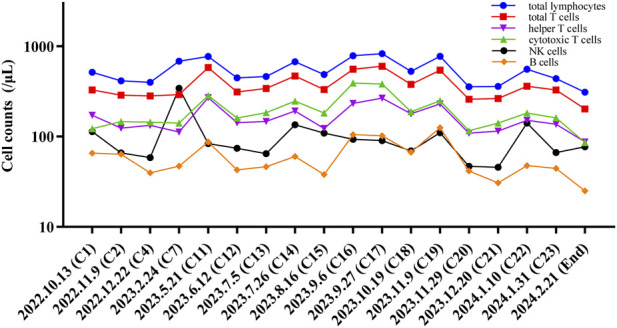
Dynamic changes in absolute counts of peripheral blood lymphocyte subsets during treatment with toripalimab and onatasertib.

**FIGURE 4:**
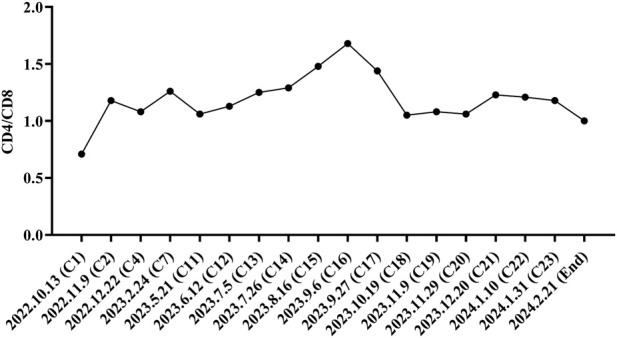
CD4/CD8 ratio increased after the treatment.

Flow cytometry was used to monitor changes in peripheral blood immune cell subsets at the start of each treatment cycle. The first sampling time point was at cycle 1 (13 October 2022, prior to any treatment), serving as the baseline. The longitudinal changes in absolute counts of six lymphocyte subsets are shown in Figure 3, and the CD4/CD8 ratio over time is presented in Figure 4.

At baseline (cycle 1), absolute counts were: total lymphocytes (CD45^+^) 515 cells/μL, total T cells (CD3^+^) 328 cells/μL, helper T cells (CD3^+^CD4^+^) 122 cells/μL, cytotoxic T cells (CD3^+^CD8^+^) 172 cells/μL, B cells (CD19^+^) 65.5 cells/μL, and natural killer (NK) cells (CD3^−^CD16^+^CD56^+^) 113 cells/μL. After 3 cycles of toripalimab and onatasertib (i.e., at the start of cycle 4), a marked increase in NK cells was observed, reaching 342 cells/μL at the start of cycle 6 (a 3.0-fold increase from baseline). In contrast, cytotoxic T cells remained low at the start of cycle 6 (112 cells/μL).

Following onatasertib dose reduction from 15 mg/kg to 10 mg/kg after cycle 11, a progressive expansion of T-cell subsets was noted. By the start of cycle 16, total lymphocytes peaked at 826 cells/μL, total T cells 600 cells/μL, helper T cells 382 cells/μL, cytotoxic T cells 266 cells/μL, B cells 102 cells/μL, and NK cells 90.2 cells/μL. The CD4/CD8 ratio, which was 0.71 at baseline, increased to 1.68 at the start of cycle 16 (Figure 4). After cycle 18, counts showed fluctuations with a general downward trend. These findings suggest that the efficacy of anti-PD-1 and mTOR inhibitor combination therapy may be associated with the expansion of helper and cytotoxic T cells, as well as NK cells.

Due to the single-patient nature of this report, no statistical comparisons were performed; the data are presented descriptively to illustrate temporal trends.

## 3 Discussion

### 3.1 Current treatment landscape and the unmet need in R/M-NPC

For patients with locally recurrent NPC, salvage surgery or re-irradiation may be options. However, it is important to note that the majority of locally recurrent NPC cases are not amenable to local treatment. The current standard first-line treatment for unresectable R/M NPC involves gemcitabine-cisplatin chemotherapy, which can be further optimized in terms of short-term efficacy by combining it with PD-1 monoclonal antibodies (Tan et al., 2022; Yang et al., 2023; Yang et al., 2021b). However, there is currently a lack of standardized salvage treatment options available for patients who have failed first-line platinum-containing regimens. Several studies have suggested that combining capecitabine, docetaxel, or vinorelbine with gemcitabine can be effective as a salvage treatment following the failure of initial platinum-based chemotherapy regimens (Chen et al., 2012; Chua et al., 2003; Ngeow et al., 2011; Zhang et al., 2008). In the realm of second-line therapy, two randomized controlled trials have been conducted to compare the efficacy and safety of PD-1 monoclonal antibodies as single agents with those of chemotherapy regimens. The KEYNOTE-122 trial revealed that pembrolizumab did not significantly enhance OS relative to chemotherapy in participants with platinum-pretreated R/M NPC (Chan et al., 2023). Similarly, another phase II randomized controlled study (NCT02605967) reported that spartalizumab was not superior to chemotherapy in platinum-refractory, R/M NPC (Even et al., 2021). The study revealed a median PFS of 1.9 months in the spartalizumab group compared to 6.6 months in the chemotherapy group (*P* = 0.915). These findings underscore the ongoing importance of chemotherapy in the management of R/M NPC in the context of second-line therapy. In light of these findings, the current guidelines recommend chemotherapy as a Grade I option for second-line treatment, indicating that PD-1 monoclonal antibodies have yet to surpass chemotherapy in terms of efficacy and safety in this setting. Currently, there is no universally accepted standard treatment for R/M NPC beyond third-line therapy.

Nevertheless, two registered studies revealed that PD-1 monoclonal antibody treatment can be effective for patients who have undergone at least two lines of chemotherapy. The POLARIS-02 study demonstrated that toripalimab treatment resulted in an objective response rate (ORR) of 23.9% among 92 patients who failed at least two lines of systemic chemotherapy, with a median PFS and OS of 2 and 15.1 months, respectively (Wang et al., 2021). Similarly, in the CAPTAIN study, camrelizumab-treated patients (n = 156) achieved an ORR of 28.2% and a median PFS and OS of 3.7 and 17.4 months, respectively (Yang et al., 2021a). Based on these promising results, the National Medical Products Administration (NMPA) has approved toripalimab and camrelizumab for the treatment of R/M NPC patients who have previously failed second-line therapy or above. These findings suggest that PD-1 monoclonal antibodies may be valuable therapeutic options for patients with R/M NPC who have exhausted other treatment alternatives. In this case, PD-1+tegafur/gimeracil/oteracil treatment resulted in a 14.6-month PFS in this patient who had failed the previous 2 lines of chemotherapy. Unfortunately, this patient ultimately developed resistance to immunotherapy. Currently, there are no biomarkers available to predict acquired resistance to PD-1 treatment, and effective treatment options for such patients are lacking.

### 3.2 Comparison with other combination strategies to overcome immune resistance

Combined treatment therapy is a good strategy for overcoming acquired resistance to PD-1 treatment. For example, ipilimumab plus anti-PD-1 therapy has yielded better results in patients with metastatic melanoma resistant to anti-PD-1 monotherapy (Pires da Silva et al., 2021). Other studies have explored the efficacy of combining radiotherapy with immunotherapy, but the results have been mixed (Schoenfeld et al., 2022; Theelen et al., 2021). In R/M NPC patients, the combination of camrelizumab and famitinib has shown promising results, with a median PFS of 7.2 months and an ORR of 33.3% (Ding et al., 2023). However, toxicities and the lack of predictive biomarkers remain challenges. Compared with these strategies, dual mTORC1/2 inhibition offers a distinct mechanism by directly targeting the PAM pathway, which is frequently hyperactivated in NPC and drives immune evasion. In this case, combined treatment with onatasertib and PD-1 inhibitor achieved a surprising nearly CR following the failure of the previous 2 lines of chemotherapy + PD-1 therapy and a PFS of 16 months, suggesting that mTOR inhibition may re-sensitize tumors to PD-1 therapy even after acquired resistance. This is consistent with preclinical evidence showing that mTOR inhibitors can reduce PD-L1 expression, reverse T-cell exhaustion, and reshape the tumor immune microenvironment (Lastwika et al., 2016; Wang Y. et al., 2024). This case provides an important platform for exploring combination therapy based on anti-PD-1 and mTOR inhibitor therapy to overcome acquired resistance to immunotherapy in R/M NPC.

### 3.3 Mechanistic insights: how onatasertib may overcome immunotherapy resistance

The PAM pathway plays a dual role in immune evasion. In cancer cells, hyperactivation of PAM signaling upregulates PD-L1 at both transcriptional and post-translational levels (Lastwika et al., 2016). In addition, mTORC1 can induce other immune checkpoints such as B7-H3/CD276, which suppresses cytotoxic T-cell activity and promotes an immunosuppressive tumor microenvironment (Liu et al., 2023). Beyond tumor-intrinsic effects, PAM signaling directly modulates T-cell function. CD28 co-stimulation enhances glycolysis via PAM, whereas PD-1 and CTLA-4 inhibit this metabolic reprogramming, leading to T-cell exhaustion (Wang S. et al., 2024). Persistent mTOR activation skews CD8^+^ T cells toward a terminally differentiated, exhausted phenotype (Huang et al., 2020). Thus, dual mTORC1/2 inhibition may reverse resistance through multiple mechanisms: (i) reducing PD-L1 and other checkpoint molecules on tumor cells, (ii) restoring cytotoxic T-cell metabolic fitness and effector function (Langdon et al., 2018; Ando et al., 2023), and (iii) decreasing immunosuppressive cells such as myeloid-derived suppressor cells and regulatory T cells (Liu et al., 2023; Huang et al., 2020).

In this case, peripheral blood immune monitoring revealed a striking expansion of NK cells at the start of cycle 7 (3.0-fold increase from baseline), followed by a progressive increase in CD4^+^ and CD8^+^ T cells after dose reduction, with the CD4/CD8 ratio rising from 0.71 to 1.68. Although these data are descriptive, they suggest that onatasertib may enhance both innate (NK) and adaptive (T-cell) immunity (Saparbay et al., 2020). The temporal correlation between T-cell expansion and sustained near-CR supports the hypothesis that dual mTORC1/2 inhibition re-invigorates antitumor immunity (Langdon et al., 2018). Notably, the NK cell surge occurred before the T-cell expansion, implying that innate immune activation might precede adaptive responses—a phenomenon observed in some preclinical models of mTOR inhibition (Liu et al., 2023).

### 3.4 Comparison with other mTOR inhibitor studies in NPC

In NPC, the mTOR pathway is among the most highly activated signaling pathways (Gong et al., 2024). A recent study showed that BRD7, a tumor suppressor frequently downregulated in NPC, inhibits PD-L1 expression via suppressing the PI3K/AKT/mTOR/STAT3 axis (Guo et al., 2025). This further emphasizes the relevance of targeting this pathway. Unlike first-generation allosteric mTOR inhibitors (rapalogs) that only inhibit mTORC1 and can induce feedback activation of AKT (O'Reilly et al., 2006), second-generation ATP-competitive mTOR inhibitors such as onatasertib block both mTORC1 and mTORC2, potentially achieving more durable suppression of the pathway (Glaviano et al., 2023). Preclinical studies have demonstrated that dual mTORC1/2 inhibitors exhibit efficacy in NPC models, primarily in combination with chemotherapy (Zhang et al., 2022). However, these agents are associated with significant toxicity (Glaviano et al., 2023). Third-generation bi-steric inhibitors (e.g., RMC-5552) are emerging, offering selective mTORC1 inhibition with potent 4E-BP1 suppression, but have not yet been evaluated in NPC (Burnett et al., 2023). Notably, the combination of mTOR inhibitors with immunotherapy has shown favorable safety profiles in clinical studies (Yan et al., 2025), supporting the feasibility of our approach. Our case provides the first clinical evidence that a dual mTORC1/2 inhibitor, when combined with PD-1 blockade, can achieve a remarkable response in a heavily pretreated, immunotherapy-resistant NPC patient.

### 3.5 Limitations

Several limitations should be acknowledged. First, this is a single case report without a control group; therefore, the observed efficacy cannot be definitively attributed to onatasertib alone, although the temporal relationship between treatment initiation and tumor response strongly supports its role. Second, follow-up is ongoing, and the long-term survival outcomes as well as the durability of the near-CR remain to be determined. Third, this is a single-center experience, and the lack of multicenter validation limits the generalizability of our findings. Fourth, we did not perform comprehensive molecular or genomic analyses (e.g., whole-exome sequencing, tumor mutational burden, or circulating tumor DNA profiling) to identify predictive biomarkers of response or acquired resistance to the combination therapy. These limitations highlight the need for larger, prospective, multicenter studies to confirm the efficacy of dual mTORC1/2 inhibition combined with PD-1 blockade in patients with R/M-NPC who have developed acquired resistance to immunotherapy.

### 3.6 Future directions

The promising signal from this case warrants further investigation. Prospective phase II trials of onatasertib plus PD-1 blockade in patients with acquired resistance to prior immunotherapy in R/M-NPC are needed. Correlative studies should include pre- and post-treatment tumor biopsies for RNA-sequencing and multiplex immunohistochemistry (e.g., CD8, FoxP3, PD-L1, p-S6) to uncover resistance mechanisms and identify biomarkers of response. Dynamic monitoring of circulating EBV DNA and peripheral immune subsets (as performed here) should be validated as predictive biomarkers in larger cohorts. Preclinical patient-derived xenograft models with acquired resistance to PD-1 could test the efficacy of dual mTOR inhibition alone or in combination. Finally, the optimal timing, dose, and sequence of combining mTOR inhibitors with immunotherapy require further optimization.

### 3.7 Conclusion

In summary, this case report demonstrates that the dual mTORC1/2 inhibitor onatasertib, when combined with toripalimab, can overcome acquired resistance to PD-1 blockade in a patient with R/M-NPC, leading to a near-CR and a 16-month PFS. The observed immune cell dynamics suggest restoration of antitumor immunity. These findings provide a strong rationale for further clinical evaluation of dual mTOR inhibition as a strategy to reverse immunotherapy resistance in NPC and potentially other PAM-driven malignancies.

## 4 Methods

Flow cytometric analysis was conducted on an Agilent NovoCyte flow cytometer using the CD3/CD8/CD45/CD4 and CD3/CD16^+^CD56/CD45/CD19 detection kits (Agilent). The 10× hemolysin was diluted 10-fold with purified water to obtain a 1× working solution. After tube labeling, 20 µL of each antibody cocktail and 50 µL of thoroughly mixed anticoagulated peripheral blood were added. The samples were gently vortexed for 5 s and incubated for 15 min at room temperature in the dark. Subsequently, 450 µL of 1× hemolysin was added, followed by another gentle vortex for 5 s and a 15-min incubation in the dark to allow complete red blood cell lysis. Data acquisition and instrument calibration were performed using NovoExpress software. The gating strategy was as follows: doublets and debris were first excluded, and lymphocytes were gated based on CD45/SSC. CD3^+^ cells were identified as T lymphocytes, which were further subdivided into CD4^+^ and CD8^+^ subsets. Among CD3^−^ cells, CD19^+^ cells were defined as B lymphocytes, and CD16^+^CD56^+^ cells as NK cells. As this is a single-patient case report, each time point was analyzed once due to limited blood volume, but instrument daily performance was verified with calibration beads.

## Data Availability

The original contributions presented in the study are included in the article/Supplementary Material, further inquiries can be directed to the corresponding authors.
