# Supplementary material for: Onatasertib re-sensitizes refractory nasopharyngeal carcinoma to immunotherapy: a Case Report
Source: Front Pharmacol. 2026 Jul 17;17:1614224. doi: 10.3389/fphar.2026.1614224 (PMC13424300; doi:10.3389/fphar.2026.1614224)
Supplement: Supplementary file 1 [file Table1.docx]

Supplementary Material

# Supplementary Table

**Supplementary Table 1.** Summary of the patient’s body weight changes and corresponding adverse event grades from baseline through the end of treatment.

| Adverse event related to weight loss | |
| --- | --- |
| Baseline level | 70 kg |
| cycle 12 (2023.6.12) | 62 kg (grade 2) |
| cycle 13 (2023.7.5) | 62 kg (grade 2) |
| cycle 14 (2023.7.26) | 62 kg (grade 2) |
| cycle 15 (2023.8.16) | 59 kg (grade 2) |
| cycle 16 (2023.9.6) | 61 kg (grade 2) |
| cycle 17 (2023.9.27) | 62 kg (grade 2) |
| cycle 18 (2023.10.19) | 63 kg (grade 2) |
| cycle 19 (2023.11.9) | 66 kg (grade 1) |
| cycle 20 (2023.11.29) | 66 kg (grade 1) |
| cycle 21 (2023.12.20) | 65 kg (grade 1) |
| cycle 22 (2024.1.10) | 64 kg (grade 1) |
| cycle 23 (2024.1.31) | 64 kg (grade 1) |
| end of study visit (2024.2.21) | 59 kg (grade 2) |
